# Paediatric Hospitalised Immune Thrombocytopenia in Real-Life Recent Viral Infection Outbreaks: A Retrospective Study

**DOI:** 10.3390/life15111736

**Published:** 2025-11-12

**Authors:** Cristina Emilia Ursu, Margit Șerban, Cristian Marius Jinca, Estera Boeriu, Ioana Ionita, Daniel Coriu, Melen Brînză, Ciprian Tomuleasa, Teodora Smaranda Arghirescu

**Affiliations:** 1 Onco-Haematology Research Unit, Romanian Academy of Medical Sciences, Children’s Emergency Hospital “Louis Turcanu” Timișoara, European Haemophilia Treatment Centre, 300011 Timișoara, Romania; emiliaursu@gmail.com (C.E.U.); mserban@spitalcopiitm.ro (M.Ș.); 2 Department of Paediatrics, Division of Onco-Haematology, “Victor Babes” University of Medicine and Pharmacy Timișoara, 300041 Timișoara, Romania; estera.boeriu@umft.ro (E.B.); sarghirescu@yahoo.com (T.S.A.); 3 Department of Haematology, “Victor Babes” University of Medicine and Pharmacy Timișoara, 300041 Timișoara, Romania; ioana.ionita@umft.ro; 4 Haematology (Clinic and Laboratory) Discipline-Fundeni Clinical Institute, “Carol Davila” University of Medicine and Pharmacy, 020021 Bucharest, Romania; daniel.coriu@umfcd.ro; 5 Department of Haematology and Bone Marrow Transplant, Fundeni Clinical Institute, 022328 Bucharest, Romania; melen.brinza@yahoo.com; 6 Department of Haematology, Research Center for Functional Genomics and Translational Medicine, Iuliu Hațieganu University of Medicine and Pharmacy, 400012 Cluj Napoca, Romania; ciprian.tomuleasa@umfcluj.ro

**Keywords:** children, immune thrombocytopenia, pandemic, viral infection, viral outbreaks

## Abstract

Immune thrombocytopenia (ITP) is an autoimmune disorder often triggered by prior viral infections. Although there is considerable evidence suggesting platelets act as passive bystanders during viral illnesses, they are increasingly recognised as active participants in their interactions with viruses. Our descriptive observational retrospective study, conducted at a tertiary hospital in Romania, aimed to evaluate the impact of viral infections on the incidence and outcomes of ITP. This cohort study focused on newly diagnosed ITP cases over a 10-year period, comparing two patient groups: the first group (I-306 patients) from the viral outbreak period (2020–2024) and the second (II-213 patients) from an epidemic-free period (2015–2019). Despite non-pharmacological measures implemented to prevent respiratory infection spread, the number (*p* = 0.05) and incidence (*p* = 0.001) of newly diagnosed ITP cases in hospitalised children increased significantly, mainly associated with severe acute respiratory syndrome coronavirus 2 (r = 0.967, *p* = 0.007), Epstein–Barr virus (r = 0.974, *p* = 0.004), and influenza (r = 0.901, *p* = 0.037), with mild thrombocytopenia (*p* = 0.028). The severity and persistence or chronicity of ITP remained unchanged. The rise in newly diagnosed ITP cases, even without increased severity or chronic evolution, may impose a substantial burden of medical and non-medical costs, highlighting the need for preventive measures during risky viral infection outbreaks.

## 1. Introduction

Although thrombocytopenia is one of the most common haematologic conditions in paediatric patients, differentiating between immune thrombocytopenia (ITP) and other causes of low platelet counts can be very challenging. ITP is an acquired autoimmune disorder, characterised by its isolated low platelet count (less than 100,000/μL), with an incidence of 1.6–3.9/100,000/year in adults and 5–10/100,000/year in children in Europe. It is a heterogeneous disease and can be categorised into primary forms, without an underlying cause, and secondary forms [1,2,3,4]. The primary isolated ITP, prevalent in childhood (more than 80% of cases), lacks an obvious predisposing or precipitating condition; therefore, it is accepted as a diagnosis of exclusion. In contrast to this type of ITP, a large number of causes, including autoimmune diseases, lymphoid malignancies, therapeutic agents, and vaccines, as well as some primary immunodeficiencies (PIDs), are determinants of secondary ITP.

Considering the timing and persistence of thrombocytopenia, ITP may be newly diagnosed (the acute form, recovering within 3 months), persistent (lasting 3–12 months), or chronic (evolving beyond 12 months). In children, approximately 25% of patients belong to the last two groups. Regarding the severity of platelet deficiency, there are three categories of ITP: severe, with at least 20,000 platelets/μL; moderate, between 20,000 and 50,000 platelets/μL; and mild, between 50,000 and 100,000 platelets/μL [1,2,3]. A lower platelet count (below 150,000/μL) is a common finding during many viral infections, suggesting that platelets may be considered passive, innocent bystanders during these infections. However, in real thrombocytopenia (under 100,000/μL), platelet count has sometimes been found to be a marker of disease severity in certain viral diseases [5,6].

It is worth mentioning the involvement in triggering secondary ITP by certain infections such as Epstein–Barr virus (EBV), hepatitis C virus (HCV), and human immunodeficiency virus (HIV), which tend to have a chronic course. Moreover, an increasing number of studies are now also revealing the association between acute respiratory or gastrointestinal viruses and the development of ITP. A high proportion of patients develop ITP following such infections, leading to a seasonal pattern of newly diagnosed ITP [2,7,8].

Furthermore, the viral infections are increasingly recognised as active participants in the complex relationship between platelets and the immune system [9]. Thrombocytopenia in response to viral infection is generally multifactorial. Viruses can modulate platelet production at various stages of development, such as altering thrombopoietin (TPO) liver production [human herpes virus 6 or 7 (HHV 6 or 7)], causing liver tissue destruction (HCV), or interfering with megakaryocytes by influencing their proliferation or activity (HCV, HIV, Cytomegalovirus—CMV). While hypo-proliferative thrombocytopenia, caused by decreased platelet production, tends to develop in the later stages of infection, some viruses induce early and rapid thrombocytopenia by promoting platelet destruction. There are many mechanisms of action: viruses can directly bind to toll-like receptors (TLR2 or TLR7), collagen receptor glycoprotein Ia/IIa (GPIa/IIa), fibrinogen receptor glycoprotein IIb/IIIa (GPIIb/IIIa), complement receptor 2 (CR2), or alter co-receptor CXCR4. Influenza virus, rhinovirus, and CMV stimulate the up-regulation of cytokines in target cells, such as Interleukin 6 (IL-6), which triggers platelet activation and reduces platelet lifespan. Moreover, it has been shown that B lymphocytes produce antibodies against certain viruses that interfere with platelet surface integrins, such as GPIIb/IIIa or glycoprotein Ib-IX-V (GPIb-IX-V). Platelet autoantibody-induced thrombocytopenia has been described in patients with HCV, HIV, CMV, EBV, hantavirus, varicella-zoster, herpes simplex virus, and severe acute respiratory syndrome coronavirus (SARS-CoV-2) [5,6,10,11].

Recent years in Romania have been marked by a distinctive viral epidemiological context, coinciding with a rise in ITP cases, which has focused our attention and concern on this issue. Recognising that the interplay between the virus and platelets could be crucial for improving and tailoring treatment strategies, we considered this a reason for our study [3,12].

Our research succeeded in documenting the reality and extent of the viral impact on ITP by conducting a comparative retrospective study based on our real-life epidemiological data.

**Aims:** This retrospective, observational, descriptive analysis was carried out at a single tertiary hospital specialised in the diagnosis and treatment of newly diagnosed ITP in children, aiming to assess its epidemiological trends, clinico-biological expression, therapeutic approach, and outcomes in the context of recent respiratory viral outbreaks. It was conducted as a cohort study, focusing on newly diagnosed ITP from 2015 to 2024.

The research was performed as a comparative study between two cohorts: one from the period of officially declared SARS-CoV-2 pandemic, followed by declared influenza A/B endemics, EBV, measles, and varicella infections, and a second cohort from a period without viral outbreaks. The primary aim of this analysis was to compare the incidence of newly diagnosed ITP in hospital-admitted patients within this epidemiological context, and the secondary aim was to examine the outcomes in relation to viral infections in ITP patients.

## 2. Patients and Methods

- Study population and setting

The study was performed on hospital-admitted paediatric patients with newly diagnosed ITP who had not been vaccinated against SARS-CoV-2.

We used our hospital’s database to select our patients based on the International Classification of Diseases-DRG System, considering the list of diseases that could potentially lead to thrombocytopenia. We used the term incidence of persons’ years/‰, referring to the infected hospitalised patients/1000 concomitant hospitalised persons at risk during the year.

b. Study periods and cohort definition

The study patients were divided into two cohorts:

- Cohort I of patients (306) admitted during 5 years between 2020 and 2024 (the period from 2020 to 2022 marked by officially declared SARS-CoV-2 pandemics, followed by 2023–2024 when influenza A/B endemics were declared in the country, alongside outbreaks of measles, varicella, and EBV infections).
- Cohort II of patients (213) is a control group focused on the 5 years pre-COVID-19 pandemic period from 2015 to 2019, free of declared viral outbreaks.

c. Inclusion and exclusion criteria

Considering the current definition of ITP accepted today as a diagnosis of exclusion [1,2], we established the obligatory criteria.

The inclusion criteria were:

- Patients ≤18 years of age, not vaccinated against SARS-CoV-2;
- Newly diagnosed thrombocytopenia ≤100,000/mm^3^ measured on two occasions within 48 h; and,
- Without clinical and biological signs suggesting a possible non-ITP type of thrombocytopenia.

**Figure 1:**
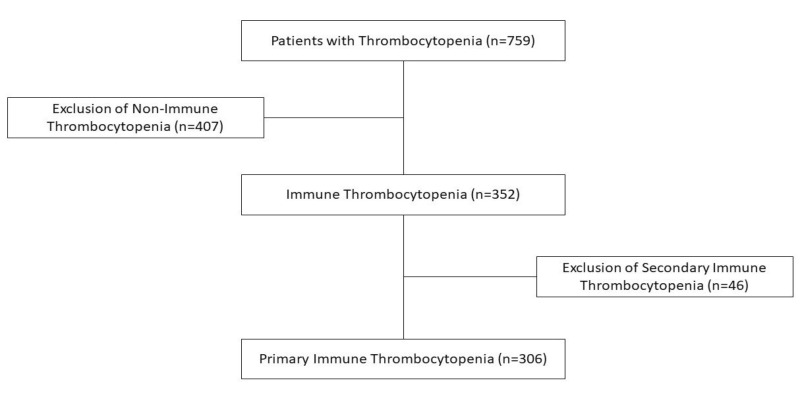
Patient inclusion/exclusion flowchart.

The exclusion criteria based on suitable exploratory data have considered persons affected by leukaemia and other oncological diseases (275), history of bone marrow transplantation or immunosuppressive therapy (17), aplastic anaemia (24), myelodysplastic syndromes (10), congenital or inherited blood disorders (5), thrombotic thrombocytopenic purpura (0), haemolytic uraemic syndrome (35), disseminated intravascular coagulation (29), vitamin B12 or folic acid deficiency (1), exposure to vaccines (4), or drugs or toxins (7) with potential haematological impact. We also excluded various immunological disorders, such as autoimmune lymphoproliferative syndrome (ALPS-2), systemic lupus erythematosus (SLE-11), antiphospholipid syndrome (APS-2), and primary immunodeficiency disorders (PID-31), all with potential pathogenic backgrounds for secondary ITP (Figure 1).

d. Diagnostic and laboratory investigations

In cohort I the following tests have been performed: a complete blood count with XN 1000-PURE RET (Alltest Biotech Co Ltd., Hangzhou, China), applying hydrodynamically focused impedance; fluorescence flow cytometry and cyanide-free SLS haemoglobin. DDS Diagnostics (RO) rapid antigen tests were used for Respiratory syncytial virus (SRV), influenza A/B, and SARS-CoV-2 viruses. Additionally, anti-SARS-CoV-2 TrimericS IgG (neutralising) antibodies were measured by chemiluminescence (CLIA) technology for the quantitative detection of antibodies against the native trimeric spike protein of SARS-CoV-2 [13]. The investigation has been extended to include the serological detection of other viruses potentially involved in the pathogenesis of ITP: HBs Antigen, anti-HCV antibodies, IgM and IgG VCA EBV, IgG EBNA EBV, IgM and IgG CMV, antibodies to HIV-1 and HIV-2 in human serum and plasma, as well as qualitative detection of HIV-1 p24 antigen, using COBAS e411—solid phase electrochemiluminescent immunoassay (ECLIA); antibodies against Herpes simplex virus 1/2, IgM and IgG, using chemiluminescence/EIA; antibodies against Herpes virus type 6 IgM, using an enzyme-linked immunosorbent assay (ELISA); and testing for adenovirus, norovirus, and rotavirus-Juschek rapid test—Hangzhou Alltest Biotech Co Ltd. (Hangzhou, China) [13].

Cohort II served as a control group for comparing the incidence and types of newly diagnosed ITP, considering only the complete blood count and its monitoring for this purpose.

e. Outcome measures

The incidence of newly diagnosed ITP, its severity grade, and the evolution in connection with viral infections were the main outcomes to be measured.

f. Statistical analysis

Statistical analyses were carried out using IBM SPSS Statistics for Windows, version 25.0 (IBM Corp., New York, NY, USA). Continuous variables were expressed as mean ± standard deviation (SD). Comparison of means within the same group, under different conditions, was performed using the paired Student’s *t*-test. Comparisons between two independent groups were performed using the independent samples (unpaired) Student’s *t*-test. Correlations between continuous variables were assessed using Pearson’s correlation coefficient for normally distributed data and Spearman’s rank correlation coefficient when at least one variable deviated from normal distribution. A two-tailed *p*-value < 0.05 was considered statistically significant. The assumptions of normality and homogeneity of variances were checked using the Shapiro–Wilk test and the Kolmogorov–Smirnov test. Figures were plotted using GraphPad Prism, version 10.6.0.

g. Ethical approval

The Institutional Ethical Review Board has approved our study. We waived the requirement for informed consent from each patient, as our retrospective study used anonymous data from the patients involved.

## 3. Results

During the pre-COVID-19 period (control group II, from 2015 to 2019), the total number of hospitalised patients was 89,244, of whom only 62,083 had a unique medical record number (UMRN). Among these, 213 patients were newly diagnosed with ITP (platelet count ≤ 100,000/mm^3^), indicating a frequency rate of 3.43‰ person-years (Table 1).

In the 5 years COVID-19 and Influenza A/B era—cohort I (2020–2024)—the number of hospitalised patients had significantly decreased due to strong recommendations for non-pharmaceutical interventions (NPIS) to prevent infections, such as lockdowns, school closures, masking, and patient isolation, reaching 68,105 (with 46,437 of them having UMRN). In contrast, the incidence of newly diagnosed ITP cases increased markedly (Table 2 and Table 3). A comparative analysis of the two cohorts (cohort I and cohort II) showed a significant rise in newly diagnosed ITP cases in cohort I, with a *p*-value of 0.05. Additionally, there was a notable increase in person-years ‰ incidence within the same cohort, reflected by a *p*-value of 0.001 (Table 3). Also noteworthy is the heterogeneous behaviour of platelet numbers during the 5 years of observation: significantly higher annual proportion of platelets (75.5% vs. 51.6% in the years 2023–2024 versus 2020–2022) due to the abandonment of NPIs and the persistence of SARS-CoV2; similarly, the severe form of ITP represented 22% in comparison to 8.66% in the years 2024–2025 and 2020–2022, respectively.

The distribution of ITP cases by gender or residence is presented in Table 3, with no significant differences observed. Concerning the severity grades of ITP, the proportion of patients in cohort I compared to cohort II for the severe form was not significantly different; the absolute numbers were 69 in cohort I and 50 in cohort II (*p* = 0.41). The same absence of a statistically significant difference was found for moderate forms (*p* = 0.43). However, in mild cases, the proportions were 63.4% (194) in cohort I and 51.17% (109) in cohort II, with a significant difference (*p* = 0.028) (Table 3).

Considering the outcome trends in cohort I, acute forms of the disease were predominant, affecting 88.23% of patients (270/306), with a statistically significant result (*p* = 0.001). Only 2.28% of patients (average 1.4 ± 0.89) developed a persistent form, while 9.48% (average 5.8 ± 1.92) exhibited a chronic form. Overall, 11.76% (36) of patients were identified as potential candidates for long-term specific therapy (Table 4), and the risk of chronic progression increased significantly with age (persistent, *p* < 0.019; chronic form, *p* < 0.001) (Table 5).

**Figure 2:**
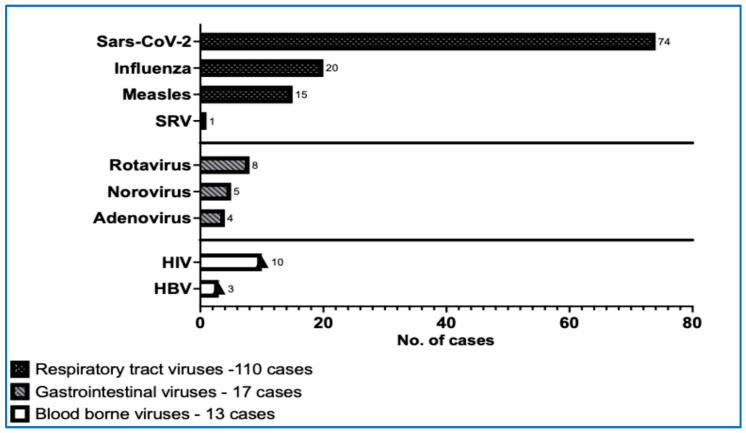
The number of viral infections in our ITP paediatric cohort I. (HBV—hepatitis B virus, HIV—human immunodeficiency virus, SARS-CoV-2—severe acute respiratory syndrome coronavirus 2, SRV—syncytial respiratory virus).

We extended the range of virological investigations in the patients of cohort I. The variety and frequency of viral infections detected in this paediatric cohort diagnosed with ITP were significantly large (Figure 2, Table 6).

In the profile of viral diseases, SARS-CoV-2 infections were the most prevalent, with a total of 74 cases reported. Among these, 28 cases were identified as current/acute infections confirmed by antigen positivity for SARS-CoV-2, while 46 cases tested positive for IgG TrimericS antibodies, indicating past infections. The next most common infection was the Epstein–Barr virus infection, with 23 patients presenting with acute or recent infections (IgM VCA positive) and 25 patients showing evidence of past infection (IgG VCA and EBNA EBV positive). Additionally, 20 patients were confirmed to have active infections of Influenza A or B through positive antigen tests. For CMV, 8 cases of recent infections and 73 cases of past infections were identified, none of which exhibited clinical symptoms. Over these five years, the profile of viral infections assessed in our ITP patients was quite extensive. Besides those previously mentioned, patients also faced infections with measles virus (15), HIV (10), rotavirus (8), varicella (9), respiratory syncytial virus (1), norovirus (5), adenovirus (4), hepatitis B (3), and herpes simplex (2) (Table 6).

**Figure 3:**
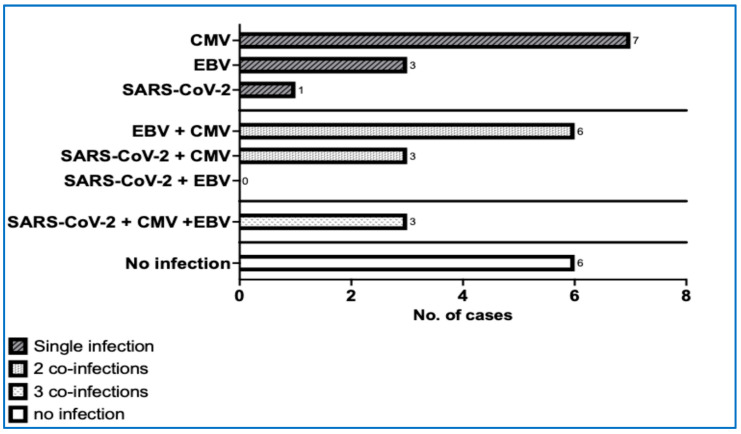
Chronic forms of ITP and the number of coinfections. (CMV—cytomegalovirus, EBV—Epstein–Barr virus, SARS-CoV-2—severe acute respiratory syndrome coronavirus 2).

The viral infections had an obvious impact on developing ITP (r = 0.916; *p* = 0.029). The correlational analysis between the total number of newly diagnosed ITP cases each year (2020–2024) and the number of infections revealed a strong correlation (r = 0.926, *p* = 0.029). ITP was mainly triggered by SARS-CoV-2 (r = 0.96, *p* = 0.007), influenza (r = 0.90, *p* = 0.037), and persistent or past EBV (ρ = 0.974, *p* = 0.004) (Table 6 and Table 7). Remarkably, chronic forms of ITP were associated with viral coinfections in 79.31% of the cases, and in 10.34% of them, even with three viruses in the same patient (Figure 3).

Regarding the effects of acute infections, we found no significant differences in the results. However, within the overall group, a notable correlation was identified between the total number of viral infections and the number of patients with thrombocytopenia, based on the chronic group (Table 8).

Regardless of the type of ITP, the clinical manifestations were mild (petechiae, posttraumatic haematoma), occurring in 55 cases; more severe bleeding (epistaxis, bruises, haemorrhagic bullae in the oral cavity) was seen in only 73 cases. In 58.16% of cases, mainly involving individuals with delayed onset of infection expression in relation to chronic or past infections, patients were asymptomatic, and thrombocytopenia was discovered during routine investigations of patients admitted for various conditions, mainly pulmonary infections, rhinosinusitis, and otitis.

The specific treatment was customised based on the platelet count, bleeding phenotype, and type of ITP (acute, persistent, or chronic). It was initiated in 73 cases presenting with severe bleeding symptoms of ITP (4 cases with moderate thrombocytopenia with platelets < 35,000/mm^3^, and 69 cases with severe thrombocytopenia), as follows: platelet concentrate in 3 cases, steroids in 38 cases, intravenous human immunoglobulin alone in 11 cases and in combination with steroids in 5 cases, and thrombopoietin receptor agonists (TPO-RAs) have been used in 16 cases (Table 9).

## 4. Discussions

The topic of thrombocytopenia in viral infections has been increasingly studied over many decades, with the past 10 years revealing numerous new insights. The recent COVID-19 pandemic has emphasised platelet-virus interactions, with many publications focusing mainly on the adult population [14,15,16,17,18] and more recently extending to the paediatric population [19,20,21,22,23,24,25,26]. Data from the Centers for Disease Control and Prevention (CDC) show that, aside from coronaviruses, the most common circulatory viruses in 2025 have been influenza, respiratory syncytial virus, and norovirus (quaddemic) [27,28].

Platelets are increasingly considered as active contributors to the antiviral immune response, interacting with both innate and adaptive immune systems, as well as directly with viruses. Various direct interactions between platelets and viruses are described: viruses can activate platelets, leading not only to enhanced platelet clearance but also to the removal of viral particles. The activated platelets can interact with B and T lymphocytes, as well as monocytes and neutrophils, ultimately improving virus neutralisation. The interplay between viruses and platelets is a highly intriguing area that is also significant from a therapeutic perspective and warrants further research [4,5,9,11].

A more common and well-recognised aspect is the quantitative impact of viruses on platelets. Thrombocytopenia as a response to viral infection is the most prevalent feature of this complex interaction [29,30,31,32]. While thrombocytopenia caused by reduced platelet production is observed at later stages of infection, viruses can also induce platelet destruction, leading to rapid-onset thrombocytopenia. In our study, thrombocytopenia was detected early during infection in 43% of cases, mainly due to SARS-CoV-2, EBV, or influenza. In contrast, thrombocytopenia was observed in 58.16% of cases among asymptomatic and oligo-symptomatic patients [30,33].

Remarkably, in our cohort I, the number of patients diagnosed in the post-COVID-19 period (2023–2024) was higher than in the previous years of officially declared SARS-CoV-2 pandemic (2020–2022). We presume that the strong preventive non-pharmacological intervention [14] effectively protected children during the declared pandemic period, while recognising the reality of the virus’s persistence in the subsequent years.

It is widely accepted that ITP in children is mainly acute and resolves spontaneously [33,34]. Even after extensive follow-up, children with chronic ITP still show a high remission rate [35]. The clinical presentation is minor in more than 77.3% of cases (petechiae, ecchymosis) and mild in 22.7% (epistaxis, dental bleeding) [36].

However, it is also essential to consider thrombocytopenia, which can be asymptomatic in hospitalised patients with various infectious diseases, as revealed also in our experience [17,21]. Such cases are also reported in other studies [5,17]. None of our patients, and very rarely in the general paediatric ITP population, progressed to significant bleeding and also to severe multisystem inflammatory syndrome (MIS), complement–mediated thrombotic microangiopathy (TMA), or thromboembolic events [1,6,7,8,12,15].

Regarding the risk of chronic evolution, the published data are very heterogeneous. ITP generally has a benign course in paediatric patients, with 86% of them fully recovering within 1 year of diagnosis [37]. In our study, 9.84% and 2.28% experienced a chronic and persistent evolution, respectively. However, the range of recovery rates is wider, varying from 5.3% to 24.4% [24,36,37]. This variation could depend on the follow-up duration: longer follow-up might show that at 2 and 5 years after diagnosis, the chances of complete remission of chronic ITP increase with 50% and 76%, respectively [36]. According to data from the Paediatric and Adult Registry on Chronic ITP, 70% of patients experienced spontaneous remission within 6 months, and 28% achieved remission within 24 months [33,34].

Considering the importance of awareness regarding the tendency of paediatric ITP evolution for the therapeutic approach, many studies have begun to evaluate clinical or laboratory predictive factors for chronic progression. It was found that increasing age, female gender, and insidious onset of symptoms are more frequently associated with the chronicity of ITP [26,36,38]. Also, in our experience, age was a decisive factor, with the median age being significantly higher in the chronic form. Other similar studies have also demonstrated that older age at diagnosis, female gender, longer duration of symptoms at diagnosis, and extended recovery time from ITP are strong predictors of chronic progression [26]. Additionally, it is suggested that a higher number of platelets at onset could also have a negative impact [38].

Viruses can influence platelet production at various stages of development; they may interfere with TPO production or replicate within megakaryocytes, impacting their proliferation and function; they can also increase apoptosis and reduce the maturation and ploidy of megakaryocytes. The range of viral causes potentially associated with ITP is large: blood-borne viruses (hepatitis B or C, human immunodeficiency virus), gastrointestinal viruses (enterovirus, rotavirus), herpesviruses (cytomegalovirus, EBV, human herpesvirus 6, varicella-zoster virus), respiratory viruses (adenovirus, influenza virus, respiratory syncytial virus, SARS-CoV-2, measles virus) [9,10,22,29].

They could be active factors capable of triggering a higher incidence of paediatric ITP, usually mild, oligo- or asymptomatic forms. In our study, 79.3% of chronic ITP cases involved multiple concurrent infections, with 13% involving even three simultaneous viral infections. These types of infections did not have a deleterious impact on increasing the proportion of severe forms. Noteworthy, however, are the observations made on 1087 patients with chronic ITP in adults [39]. Within their patient cohort, the researchers observed a significantly increased susceptibility to infections, mainly of viral origin, in the five years before ITP diagnosis; the rates of upper respiratory infections, skin infections, sexually transmitted diseases, urinary tract infections, gastrointestinal diseases, and the use of antivirals and antibiotics were also statistically higher than in individuals after ITP diagnosis. These findings may suggest a deficiency in immune reactivity, unrelated to the immunomodulatory effects of certain treatments.

ITP in children triggered by viral infections typically remains a primary disease, with a chronicity rate of less than 10–25%, unlike in adults [38]. As it lacks a specific diagnostic laboratory test, in chronic forms of paediatric ITP, it is essential to expand investigations, mainly with immunological or even genetic testing to exclude secondary PID or congenital thrombocytopenia [1]. Fortunately, in paediatric ITP, using steroids, intravenous immunoglobulin, and TPO agonists can lead to a less severe progression of the disease, avoiding the need for additional drugs like immunosuppressors or even CAR-T cell therapy [40].

The limitations of our study include its nature as a single-centre, hospital-based project, which led to fewer enrolled patients. Additionally, the retrospective design of our study could be another limitation: data are extracted from patients’ files, which depend on the quality and accuracy of their records. Finally, brief follow-up periods can be challenging for interpreting persistent or chronic forms, as a longer monitoring duration is required for greater precision; otherwise, it may negatively influence the estimated proportion of chronic cases. All these factors support an optimistic outlook on paediatric ITP, with a strong recommendation for long-term follow-up.

One advantage of our study is its comparative approach, which examines two periods with markedly different epidemiological situations; the characteristics of the disease and its evolution could suggest the best therapeutic approach.

The significantly higher number of ITP cases in cohort I (306 vs. 213 in cohort II) and the greater proportion of severe cases (69 vs. 50 in cohort II) among patients with acute forms suggest that, even if there is not a noticeably higher proportion of persistent or chronic forms, the increased total number of patients still results in substantial medical and non-medical (direct and indirect) costs that must be acknowledged.

## 5. Conclusions

Our comprehensive study highlights a significantly higher incidence of paediatric ITP linked to viral infections, mainly with SARS-CoV-2, EBV, influenza and enteroviruses. Not only in the pandemic period, but even in some following years. Fortunately, these paediatric ITP cases were mild, often oligo- or asymptomatic, without significant clinical severity or a tendency to become chronic. Clearly, aside from the rising overall number of ITP cases, there is also an increase in severe cases of the disease, even without statistical significance. This situation greatly impacts both direct and indirect medical and non-medical costs, leading to a considerable financial burden on the healthcare system. This provides a strong argument for promoting preventive measures (vaccination, NPI) during outbreaks of contagious diseases.

It is advisable and of interest for us to extend our study in the future for a longer follow up of persistent and chronic forms of newly diagnosed post-SARS-CoV-2 primary ITP patients, in order to clarify its potential connection or evolution to a secondary ITP.

## Figures and Tables

**Table 1:** Newly diagnosed ITP—cohort II.

Year	2015	2016	2017	2018	2019	Total
Number of hospitalisations	17,403	17,489	17,243	17,987	19,122	89,244
UMRN	12,373	12,240	12,064	12,482	12,924	62,083
Plt. no. ˂ 100,000/mm^3^	42	55	43	37	36	213
Incidence of ITP person’s years/‰	3.39	4.49	3.56	2.96	2.78	3.43
Plt. no. = 50–100,000/mm^3^	23	24	19	16	27	109
Plt. no. = 20–50,000/mm^3^	7	15	12	15	5	54
Plt. no. ˂ 20,000/mm^3^	12	16	12	6	4	50

(ITP—immune thrombocytopenia, no.—number, Plt.—platelet, ‰—per thousand, UMRN—unique medical record number).

**Table 2:** Newly diagnosed ITP—cohort I.

Year	2020	2021	2022	2023	2024	Total
Number of hospitalisations	8607	9443	13,650	17,108	19,297	68,105
UMRN	5864	6386	9253	12,338	12,596	46,437
Plt. no. ˂ 100,000/mm^3^	32	46	77	79	72	306
Incidence of ITP persons years/‰	5.45	7.2	8.32	6.4	5.71	6.58
Plt. no. = 50–100,000/mm^3^	19	33	52	40	50	194
Plt. no. = 20–50,000/mm^3^	8	5	13	12	5	43
Plt. no. ˂ 20,000/mm^3^	5	8	12	27	17	69

(ITP—immune thrombocytopenia, no.—number, Plt.—platelet, ‰—per thousand, UMRN—unique medical record number).

**Table 3:** Comparative data of ITP cohort I and cohort II.

	Cohort I	Cohort II	*p* Value
Number of hospitalizations	68,105	89,244	0.08
UMRN	46,437	62,083	0.06
Plt. no. ˂ 100,000/mm^3^	306	213	0.05
Incidence of ITP person- years (‰)	6.58	3.43	0.001
Plt. no. = 50–100,000/mm^3^	194 (63.4%)	109 (51.17%)	0.028
Plt. no. = 20–50,000/mm^3^	43 (14.05%)	54 (25.35%)	0.43
Plt. no. ˂ 20,000/mm^3^	69 (22.55%)	50 (23.48%)	0.41
Age (years) -mean ± SD	5.33 ± 5.28	5.87 ± 5.70	0.267
Gender -male (no, %) -female (no, %)	177 (57.84%) 129 (42.16%)	109 (51.17%) 104 (48.83%)	
Residency -Urban -Rural	156 (50.98%) 150 (49.02%)	129 (60.56%) 84 (39.44%)	

(ITP—immune thrombocytopenia, no.—number, Plt.—platelet, ‰—per thousand, %—percentage, SD—standard deviation, UMRN—unique medical record number).

**Table 4:** Outcome tendencies in newly diagnosed ITP cases—cohort I.

Year	2020	2021	2022	2023	2024	Total	*p* Value
Plt. no. ˂ 100,000/mm^3^	32	46	77	79	72	306	
Acute (no./%)	27 (84.37%)	39 (84.78%)	70 (90.90%)	68 (86.07%)	66 (91.66%)	270 (88.23%)	0.001
Chronic (no./%)	5 (15.62%)	5 (10.87%)	6 (7.8%)	9 (11.39%)	4 (5.55%)	29 (9.48%)	0.423
Persistent (no./%)	0	2 (4.35%)	1 (1.29%)	2 (2.53%)	2 (2.78%)	7 (2.28%)	0.319
Persistent + chronic (no./%)	5 (15.62%)	7 (15.22%)	7 (9.09%)	11 (13.92%)	6 (8.33%)	36 (11.76%)	0.265

(no.—number, Plt.—platelet, %—percentage).

**Table 5:** Risk factors for ITP outcomes.

Total	Acute ITP	Chronic ITP	Persistent ITP
no.	270	29	7
Age -mean ± SD	4.91 ± 5.14	8.27 ± 5.19	9.57 ± 5.94
*p*-value - age acute vs. chronic ITP - age acute vs. persistent ITP - age chronic vs. persistent ITP	0.001 0.019 0.568		
Gender -male -female	160 110	15 14	2 5
Residency -urban -rural	131 139	22 7	3 4

(ITP—immune thrombocytopenia, no.—number).

**Table 6:** Profile of viral diseases in newly diagnosed ITP cases—cohort I.

Type of Virus Infection	Type of Infection	2020	2021	2022	2023	2024	Total
SARS-CoV-2	Acute	0	3	14	5	6	28
	Past infection	0	4	5	23	14	46
EBV	Acute	3	7	5	4	4	23
	Persistent/Chronic	0	0	3	19	3	25
CMV	Acute	0	2	1	1	4	8
	Past-infection	14	8	15	30	6	73
Influenza	Acute	0	1	5	6	8	20
Measles	Acute	0	0	0	0	15	15
Varicella	Acute	1	0	1	2	1	5
Roseola Infantum	Acute	1	0	0	1	0	2
SRV	Acute	0	0	0	0	1	1
Rotavirus	Acute	0	2	4	0	2	8
Adenovirus	Acute	0	0	3	0	1	4
Norovirus	Acute	0	0	1	2	2	5
Herpes simplex	Acute	1	0	0	0	1	2
HIV	Chronic	3	3	3	0	1	10
Hepatitis B Surface Antigen	Chronic	1	0	1	1	0	3
Total		24	30	61	94	69	278

(CMV—cytomegalovirus, EBV—Epstein–Barr virus, HIV—human immunodeficiency virus, SARS-CoV-2—severe acute respiratory syndrome coronavirus 2, SRV—syncytial respiratory virus).

**Table 7:** Correlation between infections and the number of ITP cases.

	Total	r	*p*-Value	ρ	*p*-Value
Platelet no. ˂ 100,000/mm^3^	306				
SARS-CoV-2	74	0.967	0.007	-	-
EBV	48	-	-	0.974	0.004
CMV	81	0.483	0.41	-	-
Influenza	20	0.901	0.037	-	-
Varicella	5	0.555	0.331	-	-
Measles	15	0.287	0.639	-	-
Roseola infantum	2	−0.248	0.688	-	-
SRV	1	0.287	0.639	-	-
Enteroviruses (adeno-, noro- and rotavirus)	17	0.705	0.183	-	-
Herpes simplex	2	-	-	−0.577	0.308
HIV	10	-	-	−0.671	0.215
HBs antigen	3	-	-	0.288	0.637
Total infections	278	0.916	0.029	-	-

(CMV—cytomegalovirus, EBV—Epstein–Barr virus, HBs antigen—hepatitis B surface antigen, HIV—human immunodeficiency virus, SARS-CoV-2—severe acute respiratory syndrome coronavirus 2, SRV—syncytial respiratory virus, r—Pearson’s correlation coefficient, ρ—Spearman’s rank correlation coefficient).

**Table 8:** Correlation between acute and persistent/chronic or past infection and ITP.

Year	Number of Acute Infections/Total Number of Patients with Thrombocytopenia	Number of Chronic Infections/Total Number of Patients with Thrombocytopenia	Total Number of Infections/Total Number of Patients with Thrombocytopenia
2020	6/32	18/32	24/32
2021	15/46	15/46	30/46
2022	34/77	27/77	61/77
2023	21/79	73/79	94/79
2024	45/72	24/72	69/72
Total	121/306	157/306	278/306
r	0.744	-	0.916
*p* value	0.150	-	0.029
ρ	-	0.900	-
*p* value	-	0.037	-

(r—Pearson’s correlation coefficient, ρ—Spearman’s rank correlation coefficient).

**Table 9:** Treatment types in patients with severe and moderate thrombocytopenia (platelets < 35,000/mm^3^)—number of patients.

Type of Treatment	ITP Form	2020	2021	2022	2023	2024	Total
Platelet concentrate	Acute	0	0	1	1	1	3
	Chronic	0	0	0	0	0	0
Steroids	Acute	5	1	6	13	7	32
	Persistent	0	0	1	1	0	2
	Chronic	0	0	1	2	1	4
Intravenous human immunoglobulin	Acute	0	2	2	5	2	11
	Chronic	0	0	0	0	0	0
Intravenous human immunoglobulin + steroids	Acute	1	0	1	3	0	5
	Chronic	0	0	0	0	0	0
TPO-RAs	Acute	0	0	0	0	0	0
	Persistent	0	0	0	0	1	1
	Chronic	3	2	3	4	3	15
	Total	9	5	15	29	15	73

(ITP—immune thrombocytopenia, TPO-RAs—Thrombopoietin receptor agonists).

## Data Availability

The original contributions presented in this study are included in the article. Further inquiries can be directed to the corresponding author.
